# Miniature Inverted Repeat Transposable Element Insertions Provide a Source of Intron Length Polymorphism Markers in the Carrot (*Daucus carota* L.)

**DOI:** 10.3389/fpls.2017.00725

**Published:** 2017-05-09

**Authors:** Katarzyna Stelmach, Alicja Macko-Podgórni, Gabriela Machaj, Dariusz Grzebelus

**Affiliations:** Faculty of Biotechnology and Horticulture, Institute of Plant Biology and Biotechnology, University of Agriculture in KrakowKrakow, Poland

**Keywords:** *DcSto*, genetic diversity structure, ILP, *Stowaway*-like MITEs, TEs

## Abstract

The prevalence of non-autonomous class II transposable elements (TEs) in plant genomes may serve as a tool for relatively rapid and low-cost development of gene-associated molecular markers. Miniature inverted-repeat transposable element (MITE) copies inserted within introns can be exploited as potential intron length polymorphism (ILP) markers. ILPs can be detected by PCR with primers anchored in exon sequences flanking the target introns. Here, we designed primers for 209 *DcSto* (*Daucus carota Stowaway*-like) MITE insertion sites within introns along the carrot genome and validated them as candidate ILP markers in order to develop a set of markers for genotyping the carrot. As a proof of concept, 90 biallelic *DcS*-ILP markers were selected and used to assess genetic diversity of 27 accessions comprising wild *Daucus carota* and cultivated carrot of different root shape. The number of effective alleles was 1.56, mean polymorphism informative content was 0.27, while the average observed and expected heterozygosity was 0.24 and 0.34, respectively. Sixty-seven loci showed positive values of Wright's fixation index. Using Bayesian approach, two clusters comprising four wild and 23 cultivated accessions, respectively, were distinguished. Within the cultivated carrot gene pool, four subclusters representing accessions from Chantenay, Danvers, Imperator, and Paris Market types were revealed. It is the first molecular evidence for root-type associated diversity structure in western cultivated carrot. *DcS*-ILPs detected substantial genetic diversity among the studied accessions and, showing considerable discrimination power, may be exploited as a tool for germplasm characterization and analysis of genome relationships. The developed set of *DcS*-ILP markers is an easily accessible molecular marker genotyping system based on TE insertion polymorphism.

## Introduction

Transposable elements (TEs) are segments of DNA that can move themselves to new chromosomal location. They are prevalent in the genomes of both prokaryotes and eukaryotes, and account for a great subsection of the genetic variation in plants and animals. Some plant genomes are composed of transposable elements in more than two thirds, as the 77% of the maize genome (Meyers et al., 2001). Miniature inverted-repeat transposable elements (MITEs) are a special type of class II non-autonomous elements with a maximum of a few hundred base pairs in size (Hua-Van et al., 2005). Although they were first discovered in plant genomes (Bureau and Wessler, 1992, 1994), they have been also identified in a wide range of animal, eubacteria and archea genomes (Brügger et al., 2002; Feschotte et al., 2002). The two largest MITE families, *Stowaway* and *Tourist*, were identified as members of the *Tc1/Mariner* and the *PIF/Harbinger* superfamilies, respectively (Jiang et al., 2004). *Stowaway* MITEs were first described in the maize genome (Bureau and Wessler, 1994) as less than 500 bp long, forming a 2 bp TA TSD upon insertion. MITEs are usually present in many thousand copies per genome. 22,000 identified *Stowaway* MITEs were classified into 34 families in the *Oryza sativa* genome (Feschotte et al., 2003), whereas 18,000 MITE insertions were classified into 18 families in the *Triticum* spp. genome (Yaakov et al., 2013).

The ubiquity, genome-wide distribution and high copy numbers have provided genetic markers from both class I and class II TEs (Kumar and Hirochika, 2001). The abundance of MITE copies makes them highly useful source of polymorphism. To date, MITE Transposon Display (MITE-TD) and Inter-MITE Polymorphism (IMP) techniques exploiting the TIR sequences in *Oryza sativa, Zea mays, Sorghum bicolor, Hordeum vulgare*, and *Daucus carota* MITEs, have been developed (Chang et al., 2001; Park et al., 2003; Casa et al., 2004; Lee et al., 2005; Grzebelus et al., 2007). Some *Stowaway* MITEs identified to date were described as being preferentially inserted or retained in genic regions (Casa et al., 2000; Jiang et al., 2003). However, even though 54% of *DcSto* insertion sites in the carrot genome were located less than 2 kb away from or inside the coding sequences, random distribution of *DcSto* rather than preferential insertions around genes was proposed (Iorizzo et al., 2016).

Insertions within introns may provide a significant polymorphism. Intron polymorphisms, particularly intron length polymorphisms (ILPs), can be exploited as genetic markers used for gene mapping (Wydner et al., 1994) and population genetic surveys (Lessa, 1992). ILP takes advantage of the different rate of evolution of exons and introns that can result in conserved exon nucleotide sequences adjoined to more variable intron sequences. ILP can be detected by the polymerase chain reaction with a pair of primers anchored in the exons flanking the intron of interest (Wang et al., 2005). ILP markers are unique due to their gene-specifity, codominancy, conveniency, reliability and cost-efficiency. Furthermore, ILPs are characterized by high transferability among related plant species (Yang et al., 2007; Gupta et al., 2011). To date, studies on the development of ILP markers in plants have been restricted to few species (Wang et al., 2005; Huang et al., 2008; Chen et al., 2010; Gupta et al., 2011, 2012; Li et al., 2013; Muthamilarasan et al., 2014).

Carrot is the most widely grown member of Apiaceae family. Its progenitor, wild *Daucus carota* L., is a plant commonly occurring in the temperate climatic zones. To date, a range molecular tools facilitating genome analysis in context of evolutionary history of wild and cultivated carrot have been developed, i.e., DArT, SSR, and SNP markers (Cavagnaro et al., 2011; Iorizzo et al., 2013; Grzebelus et al., 2014) and a set of ca. 30 resequenced genomes (Iorizzo et al., 2016). The analyses showed clear evidence for the carrot germplasm separation into three distinct groups of wild, western cultivated (European and American germplasm) and eastern cultivated (Asian germplasm) carrot. The majority of modern cultivars belong to the western group. Several varietal types were distinguished within western carrots, based primarily on the storage root shape and size (Prohens and Nuez, 2008). Despite apparent phenotypic differences, previous studies have indicated absence of any apparent population structure in western carrots, suggesting no significant genetic separation among these varietal types (Bradeen et al., 2002; Iorizzo et al., 2013).

In this study, we performed (1) a genome-wide search for *DcSto* (*Daucus carota Stowaway*-like) MITE insertion-based intron length polymorphism markers, and (2) validation of candidate ILP markers in order to develop a panel for genotyping the carrot by means of applying a simple, cost- and time-efficient polymerase chain reaction.

## Materials and methods

### Plant materials

Twenty eight carrot accessions comprising four wild carrots of different origin, 23 western type carrot cultivars representing four types of root shape and a DH1 plant (Iorizzo et al., 2016) as the reference, were used for ILP validation (Table 1). Total genomic DNA was isolated from fresh young leaves using commercial DNeasy Plant Mini Kit (Qiagen) and used as the template for PCR amplification.

**Table 1:** **Description of plant material used in the present study**.

**Number**	**Accession**	**Species**	**Cultivar name**	**Root type**	**Origin**	**Source**	
1	RS33	*Daucus carota* subsp. *sativus*	Chantenay Royal	Chantenay	FRA	HRIGRU	8860
2	RS34	*Daucus carota* subsp. *sativus*	Chantenay Red Cored	Chantenay	GBR	HRIGRU	8847
3	RS35	*Daucus carota* subsp. *sativus*	Royal Chantenay	Chantenay	USA	HRIGRU	3882
4	RS37	*Daucus carota* subsp. *sativus*	Gold King	Chantenay	USA	HRIGRU	5127
5	RS39	*Daucus carota* subsp. *sativus*	Chantenay Long Type	Chantenay	USA	HRIGRU	5090
6	RS41	*Daucus carota* subsp. *sativus*	Chantenay Rex RS	Chantenay	NLD	HRIGRU	5589
7	RS43	*Daucus carota* subsp. *sativus*	Danvers 126	Danvers	GBR	HRIGRU	6487
8	RS44	*Daucus carota* subsp. *sativus*	Danvers Danro RS	Danvers	NLD	HRIGRU	5595
9	RS45	*Daucus carota* subsp. *sativus*	Danvers Red Cored	Danvers	USA	HRIGRU	5128
10	RS49	*Daucus carota* subsp. *sativus*	Danvers	Danvers	NLD	HRIGRU	11144
11	RS50	*Daucus carota* subsp. *sativus*	Danvers Pride	Danvers	USA	HRIGRU	8098
12	RS51	*Daucus carota* subsp. *sativus*	Danvers Half Long	Danvers	USA	HRIGRU	8109
13	RS56	*Daucus carota* subsp. *sativus*	Paris Market	Paris Market	NLD	HRIGRU	5596
14	RS57	*Daucus carota* subsp. *sativus*	Paris Forcing	Paris Market	GBR	HRIGRU	3966
15	RS59	*Daucus carota* subsp. *sativus*	French Forcing Horn	Paris Market	GBR	HRIGRU	6489
16	RS60	*Daucus carota* subsp. *sativus*	Parijse Market	Paris Market	—	HRIGRU	9294
17	RS62	*Daucus carota* subsp. *sativus*	Parijse Market (Rubin)	Paris Market	—	HRIGRU	9296
18	RS71	*Daucus carota* subsp. *sativus*	Gold Pak	Imperator	USA	HRIGRU	3885
19	RS72	*Daucus carota* subsp. *sativus*	Imperator 408	Imperator	USA	HRIGRU	3907
20	RS73	*Daucus carota* subsp. *sativus*	Imperator	Imperator	NLD	HRIGRU	11145
21	RS74	*Daucus carota* subsp. *sativus*	Imperator 407	Imperator	USA	HRIGRU	3891
22	RS75	*Daucus carota* subsp. *sativus*	Long Imperator 58	Imperator	USA	HRIGRU	3917
23	RS76	*Daucus carota* subsp. *sativus*	Imperator 58	Imperator	USA	HRIGRU	3892
24	CDS15	*Daucus carota* subsp. *azoricus*	–	–	ESP	HRIGRU	6667
25	CDS39	*Daucus carota* subsp. *carota*	–	–	CHE	HRIGRU	9226
26	CDS93	*Daucus carota* subsp. *carota*	–	–	USA	USDA	–
27	CDS40	*Daucus carota* subsp. *carota*	–	–	POL	HRIGRU	9270

### Development of ILP markers

Coordinates of 4028 *DcSto* insertions belonging to 14 families were compared to coordinates of ca. 32 thousand genes annotated in the carrot reference DH1 genome assembly (Iorizzo et al., 2016; NCBI accession LNRQ01000000). 609 gene-associated *DcSto* insertion sites localized in introns were identified, of which 209 were manually selected for development of ILP markers. The criteria for initial selection were as followed: insertion sites were (1) free from any other annotated repetitive sequences, (2) present in introns not longer than 3.7 Kb, and (3) evenly distributed over each chromosome. Primer3 (Untergasser et al., 2012) and Primer-BLAST (Ye et al., 2012) were used to design PCR primer pairs anchored in exons flanking introns harboring the selected *DcSto* insertions. Primer pairs were designed to amplify fragments in a 400–3,700-bp range. The optimal annealing temperature was set to 58°C; and the size and GC content ranged from 18 to 23 bases and 40 to 60%, respectively.

### Validation and evaluation of *DcS*-ILP markers

Candidate ILP markers were selected for experimental evaluation. Amplification was carried out in a 10 μL total volume containing 20 ng of genomic DNA, 0.5 μM each of forward and reverse primer, 0.25 mM of each dNTP (Thermo Fisher Scientific), 0.5 U Taq DNA polymerase (Thermo Fisher Scientific) and 1x Taq buffer. The PCR amplifications were performed in an Eppendorf MasterCycler Gradient using the following thermal profile: 94°C (120 s), 30 cycles of 94°C (30 s), 56°C (30 s), 68°C (120 s) and final step of 68°C (600 s). For primers generating ambiguous profiles, the annealing temperature was adjusted to 58, 59, or 60°C. PCR products were separated in 1% agarose gels run in 1x Tris-borate-EDTA buffer (pH 8.0) at a constant current of 5V/cm for about 2 h, stained with Midori Green (Nippon Genetics) and analyzed using GelDoc-It imaging system (UVP). GeneRuler 1 kb and 100 bp^+^ DNA Ladders (Thermo Fisher Scientific) were used to determine product sizes for each locus. The amplicons representing additional local rearrangements within introns were excised, purified using GenJET™ Gel Extraction Kit (Thermo Fisher Scientific), cloned into T/A cloning vector (Promega Corporation) and transformed into *Escherichia coli*, strain DH10B. Up to five recombinant colonies were selected and cultured overnight at 37°C in culture tubes containing 5 mL of Luria–Bertani medium and ampicillin (100 mg/L). Plasmids were purified using Wizard SV Minipreps KIT (Promega Corporation). Sequencing reactions were set up with universal primers sp6 and T7 using Big Dye terminator chemistry (Applied Biosystems), as recommended by manufacturer. Sequencing was carried out on ABI 3700 capillary sequencer (Applied Biosystems).The sequences were manually edited using BioEdit (Hall, 1999) and aligned to the sequences of predicted genes for which ILP primers were designed.

### Recording of electrophoretic bands and statistical data analysis

The ILP marker profiles were scored manually. Each allele was scored as: 1 (empty insertion site), 2 (occupied insertion site) or 0 (lack of amplification).The codominant marker matrix with diploid individuals was created (Supplementary Table 1) and used in GenAlEx 6.5 (Peakall and Smouse, 2006) for creating genetic distance matrix and analysis of molecular variance (AMOVA). Expected and observed heterozygosity (H_*e*_ and H_*o*_), and fixation index (F_*IS*_) were computed using POPGENE 1.32 (Yeh et al., 2000). Polymorphism informative content (PIC) of *n*-allele locus, an indicator of a genetic marker's usefulness introduced by Botstein et al. (1980), was calculated as: $PIC=1-{\sum_{i=1}^{n}p_{i}^{2}}^{\text{​}}-{\sum_{i\;=\;1}^{n-1}\sum_{j\;=\;i+1}^{n}2p_{i}^{2}p_{j}^{2}}^{\text{​}}$, where *p*_*i*_ and *p*_*j*_ are the population frequency of the *i*th and *j*th allele. Genetic structure was inferred using Bayesian model-based software STRUCTURE 2.2.3 (Pritchard et al., 2008) without information on the accession origin. Ten independent iterations with an admixture and correlated allele frequencies model were performed. The length of the burn-in period and the number of Markov Chain Monte Carlo (MCMC) replications after the burn-in were assigned at 10^5^ for each number of clusters (K) set from 1 to 27 and 1 to 23 for further subclustering. The estimation of K was provided by joining the log probability of data [LnP(D)] from STRUCTURE output and an *ad hoc* statistics ΔK (Evanno et al., 2005) based on the second rate of change of the log probability of data with respect to the number of clusters. In addition, CLUMPAK software (Kopelman et al., 2015) was used to confirm the selection of the best K. Based on the chosen K, each carrot accession was assigned to a subpopulation for which its membership value (Q) was higher than 0.6. AMOVA was performed using GenAlEx 6.5 to evaluate differentiation among the subpopulations. Principal coordinate analysis (PCoA) was conducted to visualize genetic diversity of the studied accessions.

## Results

### Development and validation of the candidate ILP markers

**Figure 1:**
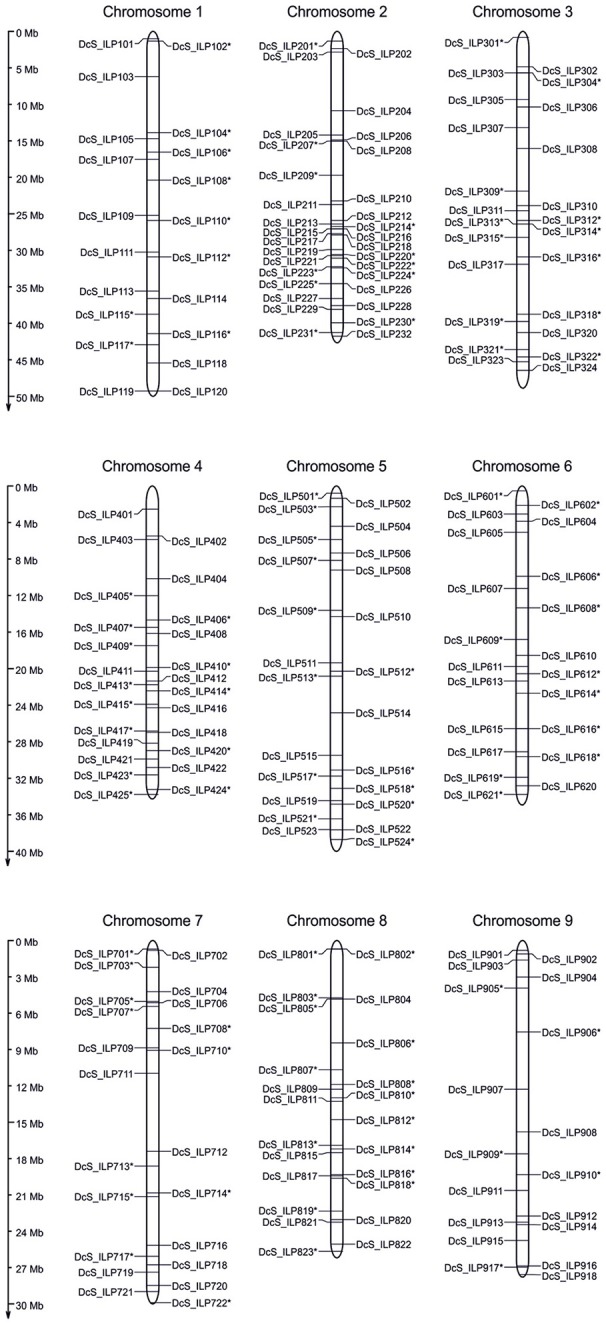
**Physical genomic distribution of the 209 developed ***DcS***-ILP markers on nine chromosomes of the carrot genome**. The vertical bars correspond to the position of introns harboring *DcSto* insertions, selected for a development of ILP markers. Positively validated markers are marked by asterisk.

Insertion sites of 209 *DcSto* MITEs within introns of annotated genes were chosen to develop *Daucus carota Stowaway*-like Intron Length Polymorphism (*DcS*-ILP) markers evenly distributed throughout the genome (Figure 1). The number of *DcSto* insertion sites evaluated per chromosome varied from 18 (chromosome 9) to 32 (chromosome 2), with an average of 23.22. Their density ranged from 1.37 (chromosome 2) to 2.57 per Mb (chromosome 1), with an average of 1.76.

**Figure 2:**
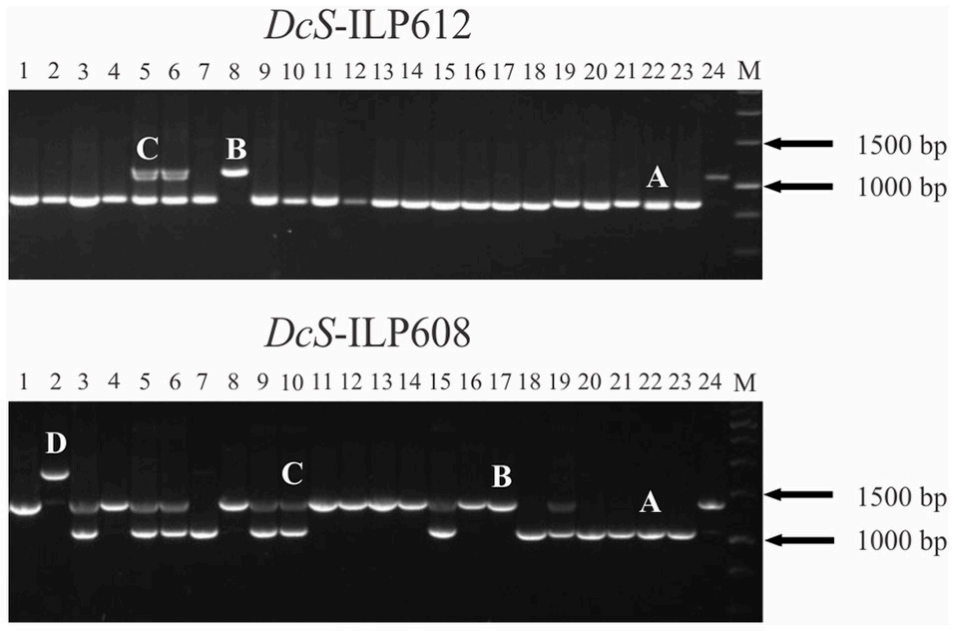
**Amplification of ***DcS***-ILP612 and ***DcS***-ILP608 markers in 23 carrot cultivars and the reference genome**. Carrot accessions from 1 to 24 are listed in Table 1. *DcS*-ILP612—amplification of two alleles corresponding to empty **(A)** and occupied **(B)** *DcSto* insertion site and heterozygote **(C)**; *DcS*-ILP608—amplification of an additional allele **(D)** resulting from an unclassified rearrangement within the intron. M, 1 kb DNA Ladder.

Upon PCR amplification, 100 of the 209 sites showed the expected *DcSto* insertion-based polymorphism, however, in case of 10 sites at least one additional amplicon was present in at least one accession (Figure 2). Sequencing of those amplicons revealed that none of the additional variants was related to the activity of the *DcSto* copy present in the reference genome (data not shown). Of the remaining 109 sites, six did not amplify efficiently; 32 were monomorphic for all tested plants; 13 showed a complex pattern resulting from nonspecific amplification, whereas 58 yielded polymorphic products not associated with *DcSto* insertions (i.e., sizes of PCR products did not correspond to the expected sizes of empty or occupied variants) (Table 2).

**Table 2:** **Results of the experimental validation of developed candidate ***DcS***-ILP markers**.

		**Validated insertion sites**					
**Chromosome**	**Number of insertion sites**	**Polymorphic with two allelic variants resulting from *DcSto* insertion**	**Polymorphic with two allelic variants resulting from *DcSto* insertion and an additional variant**	**Polymorphic with many allelic variants not associated with *DcSto* insertion**	**Complex amplification pattern**	**Monomorphic**	**No amplification**
1	20	9	–	7	1	2	1
2	32	11	–	12	2	6	1
3	24	8	4	4	3	4	1
4	25	11	2	6	1	4	1
5	24	10	3	6	1	3	1
6	21	10	1	6	1	3	–
7	22	11	–	5	1	5	–
8	23	15	–	7	–	1	–
9	18	5	–	5	3	4	1
Total	209	90	10	58	13	32	6

The length of introns harboring the selected *DcSto* insertions varied from 449 to 3,637 bp. Based on the length of amplified introns, the developed markers were divided into six classes; I to V with intron size ranging from 400 to 3,400 bp, each at 600-bp interval, and class VI comprising introns longer than 3,400 bp (Table 3). Introns belonging to classes I to IV comprised 97.6% of all the developed markers. Class I and II markers were the most numerous, whereas class III markers showed the highest (55.6%) successful amplification rate indicating the most suitable length of introns considered for ILP markers. DcS-ILP markers of class V and VI were characterized by ambiguous amplification patterns, therefore not considered for further analyses.

**Table 3:** **The intron length-based classification of candidate ***DcS***-ILP markers**.

**Marker class**	**The range of intron lengths [bp]**	**Number of candidate *DcS*-ILP markers**	**Number of positively validated *DcS*-ILP markers**
I	400–1,000	75	34
II	1,001–1,600	80	34
III	1,601–2,200	27	15
IV	2,201–2,800	22	7
V	2,801–3,400	4	0
VI	>3,401	1	0

Finally, 90 *DcS*-ILP (Supplementary Table 2) markers showing biallelic *DcSto* insertion polymorphism (Figure 2) were chosen for development of a panel for genotyping the carrot.

### Assessment of genetic diversity

The utility of 90 biallelic *DcS*-ILP markers was verified by estimating the genetic diversity of the collection of 27 *D. carota* accessions comprising 23 cultivated and 4 wild populations. In total, 180 alleles were identified with an average of 2.0 per locus. 2.78% of the alleles were rare (frequency <0.05) and the mean effective number of alleles was 1.56. The observed heterozygosity for individual loci ranged from 0.04 to 0.56, with an average of 0.24, whereas the expected heterozygosity ranged from 0.04 to 0.51, with an average of 0.34. Shannon's index was from 0.09 to 0.69, with an average of 0.50. Among all the loci analyzed with the Wright's fixation index, 67 were positive. The PIC values ranged from 0.04 to 0.37, with an average of 0.27 (Supplementary Table 1).

**Figure 3:**
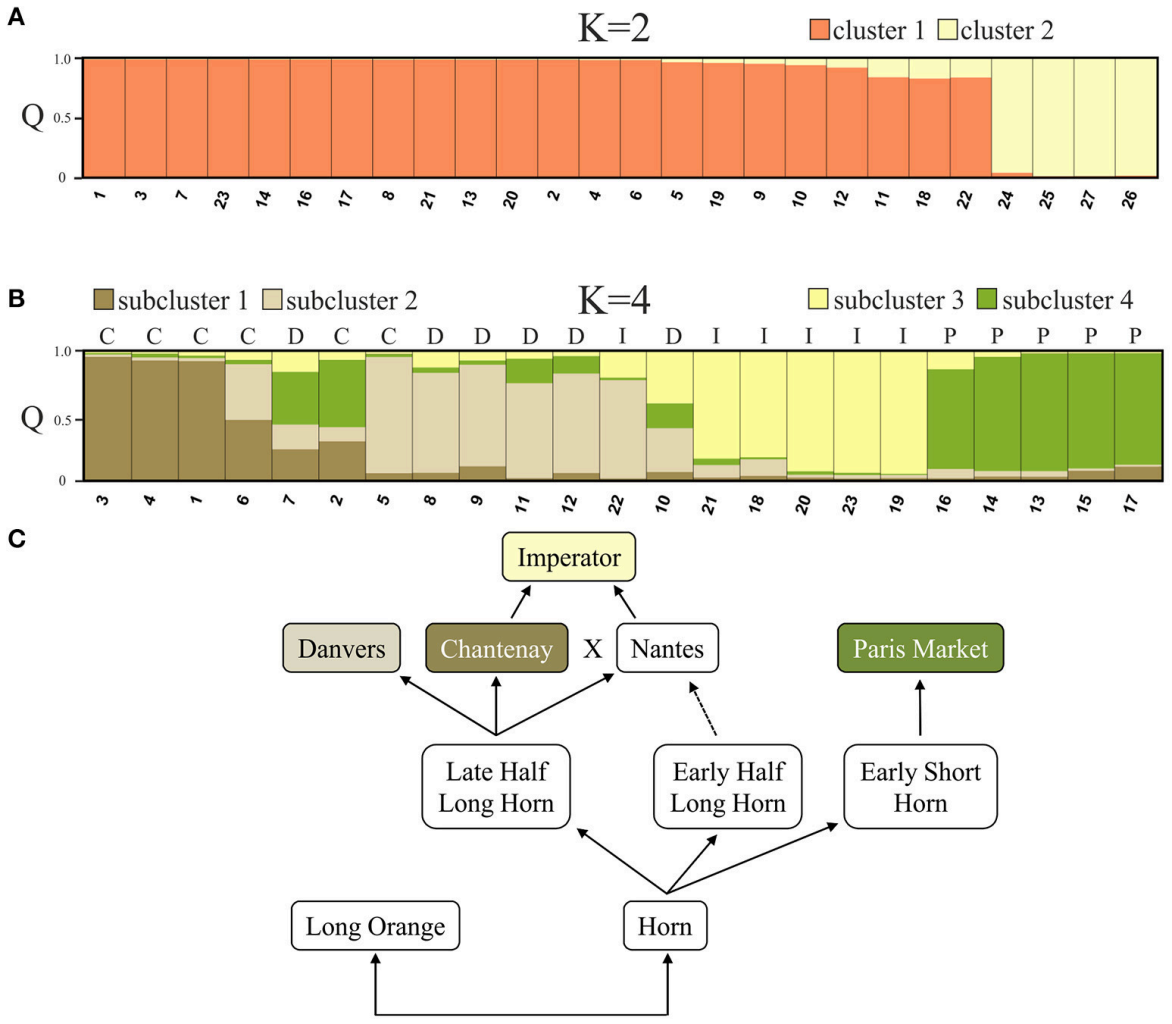
**The genetic structure of the studied 27 accessions based on a Bayesian approach assuming two clusters comprising cultivated (cluster 1) and wild (cluster 2) accessions, exclusively (A)**. The analysis of the genetic structure within first cluster resulted in forming four subclusters, generally comprising accessions representing each of described storage root shapes: C, Chantenay; D, Danvers; I, Imperator; P, Paris Market **(B)**. Assumed four gene pools reflect their breeding history as proposed by Banga (1963) **(C)**. The numbers of accessions correspond to those listed in Table 1.

STRUCTURE analysis based on 90 loci representing *DcSto* insertion-derived polymorphisms was performed to evaluate genetic structure of the 27 accessions. The value of ΔK statistics was the highest when two clusters were assumed [Δ*K*_(2)_ = 297.64]. The increase in the number of assumed clusters resulted in low Δ*K* value [Δ*K*_(>2)_ = 0.01–52.35]. Twenty three cultivated accessions were assigned to cluster 1 (C1) with membership coefficients (Q) ranging between 0.831 and 0.997, whereas cluster 2 (C2) comprised exclusively wild accessions with the *Q* value of 0.965–0.998 (Figure 3A). The level of genetic diversity within C1 (0.31) was slightly higher than within C2 (0.29).

To evaluate the genetic structure of the 23 cultivated accessions further subclustering was performed on the accessions assigned to C1. The highest ΔK was observed for *K* = 21 [Δ*K*_(21)_ = 22.77], *K* = 2 [Δ*K*_(2)_ = 17.33] and *K* = 4 [Δ*K*_(4)_ = 14.55]. Δ*K* values for *K* = 3, *K* = 5–20 and *K* = 22–23 were not significant (Δ*K* = 0.164–4.16). The mean value of log probability of the data was higher for *K* = 4 than for *K* = 21, and *K* = 2 [LnP(D)_*K* = 4_ = −1891.7, LnP(D)_*K* = 2_ = −1922.5, LnP(D)_*K* = 21_ = −2703.2], therefore four subclusters were chosen as the most probable genetic structure of the studied cultivated accessions. With *K* = 4, three accessions were assigned to subcluster SC1 with Q ranging between 0.928 and 0.962, six to subcluster SC2 with Q between 0.746 and 0.908, five to subcluster SC3 with Q between 0.825 and 0.954 and five to subcluster SC4 with Q between 0.782 and 0.922 (Figure 3B). Four accessions, namely Chantenay Red Cored, Chentenay Rex RS, Danvers 126, and Danvers could not be assigned to any of the subclusters due to high level of admixture (*Q* < 0.6). The overall Q proportion of each of the four types clearly distinguished (*Q* > 0.6) the membership of Chantenay root type in SC1 (*Q* = 0.605), Danvers root type in SC2 (*Q* = 0.626), Imperator root type in SC3 (*Q* = 0.785), and Paris Market root type in SC4 (*Q* = 0.884) (Table 4).

**Table 4:** **The proportion of membership coefficients (Q) of each population defined by the type of root in each of the four subclusters**.

**Population name**	**Q proportion for four assumed subclusters**				**Number of accessions assigned to defined population**
	**SC1**	**SC2**	**SC3**	**SC4**	
Chantenay	0.605	0.253	0.031	0.111	6
Danvers	0.082	0.626	0.136	0.155	6
Imperator	0.014	0.175	0.786	0.024	6
Paris market	0.043	0.034	0.039	0.884	5

**Figure 4:**
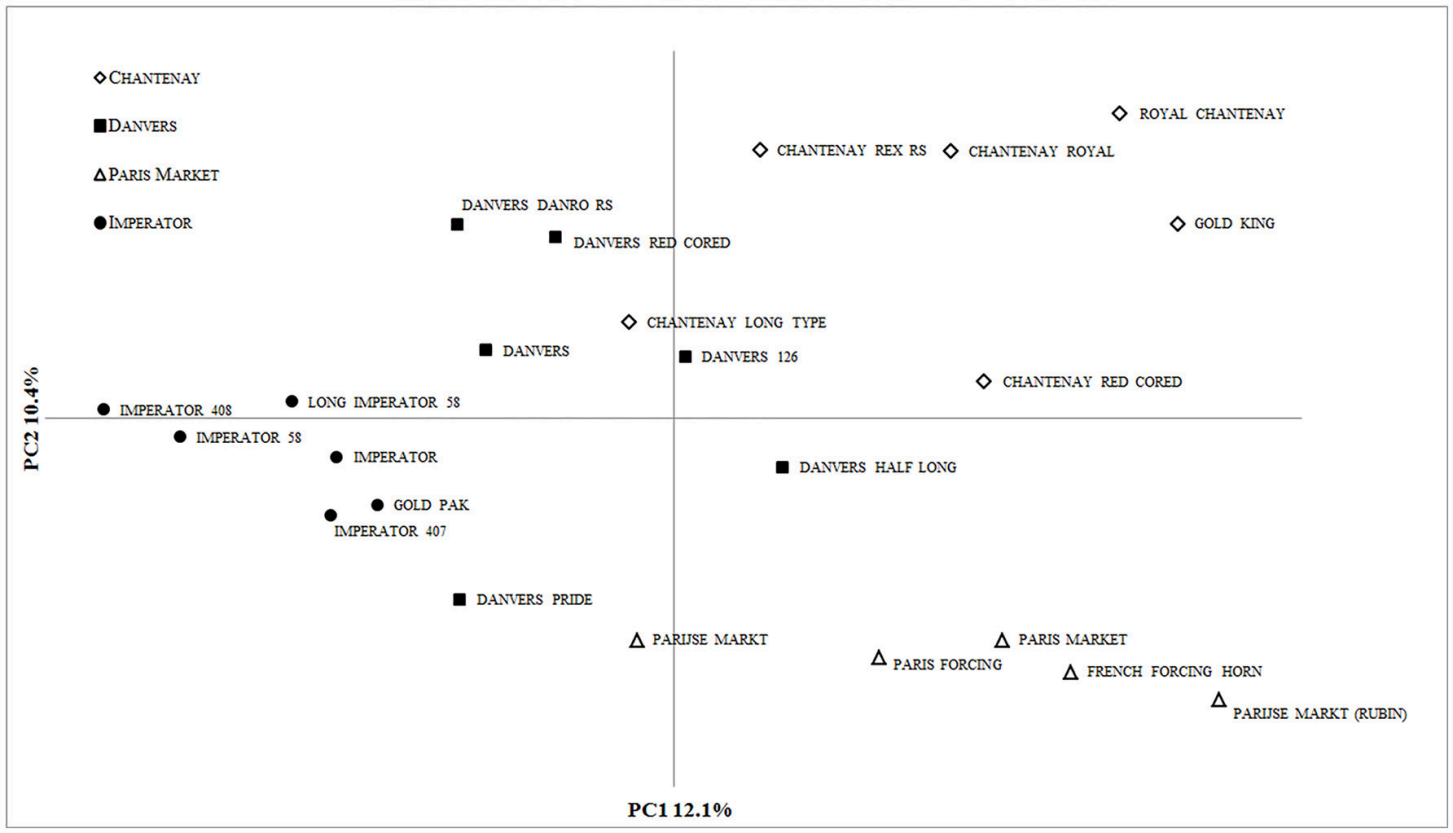
**PCoA of 23 carrot accessions based on 90 ***DcS***-ILP markers**.

AMOVA attributed 19% (*P* = 0.001) of the total genetic diversity to variation among the root types. The diversity of the 23 cultivated accessions was revealed by PCoA (Figure 4). Using the first three axes 31.7% of the total variation could be explained, with the 1st, 2nd, and 3rd axes explaining 12.1, 10.4, and 9.2%, respectively.

The above results suggested four separate groups in the collection of 23 cultivated carrots and the grouping generally corresponded with a postulated breeding history of western carrot types presented by Banga (1963), indicating that Chantenay and Danvers types originated from the Late Half Long Horn group, while Paris Market type descended from the Early Short Horn group. Both historical groups differ in terms of their storage root shape and earliness. In turn, the origin Imperator type was traced back to a cross between Chantenay and Nantes (Figure 3C).

## Discussion

In the present study, we took advantage of intron length polymorphisms resulting from retained *DcSto* insertions in order to develop a set of ILP markers in the carrot. The *DcSto* elements used in the study comprised mostly two families, *DcSto6* and *DcSto1*, the most numerous in the carrot genome and showing high percentage of insertions within coding regions (20 and 12%, respectively) (Iorizzo et al., 2016). The ubiquity of *DcSto* elements facilitated the selection of evenly distributed insertion sites for analysis, as well as equal coverage of the genome with the developed markers. 62.7% of the candidate markers were successfully amplified and 47.8% of them identified *DcSto* insertion polymorphisms. The success of amplification rate was lower in comparison with ILP markers in other plants, such as *Vigna unguiculata* (89%; Gupta et al., 2012), *Glycine max* (88.2%; Shu et al., 2010), *Solanum lycopersicum* (71%; Wang et al., 2010), probably as a result of high percentage of ambiguous amplification of introns longer than 2,200 bp. The length of intron is considered the main cause of PCR failure and generally, the successful amplification rate decreases with greater length of intron (Wang et al., 2010; Gupta et al., 2012). Polymorphism information content (PIC) has become the most widely used formula to measure the information content of molecular markers (Nagy et al., 2012). The mean PIC value of *DcS*-ILPs obtained for the studied *Daucus carota* accessions was higher compared to many of the developed ILP markers, e.g., *Setaria italica* (Gupta et al., 2011) and *Hevea brasiliensis* (Li et al., 2013), and comparable to study of Gupta et al. (2012) where 16 CILP loci were analyzed in 10 *Vigna unguiculata* accessions, with an average of 2.0 alleles per locus, and PIC value of 0.34. Differences in PIC values might be attributed to the various numbers of markers and accessions exploited in these studies. The average PIC value obtained in study of Huang et al. (2010), where 103 ILP loci were analyzed in 36 *Oryza sativa* accessions, was considerably higher (0.44) due to the higher number of alleles identified by rice ILPs (2.29 alleles per locus). As expected, the mean PIC value of the codominant *DcS*-ILPs was lower than the one obtained for the genomic SSR markers developed for the carrot (Rong et al., 2010; Cavagnaro et al., 2011). Similar results were reported for the comparative analysis of genetic diversity in *Oryza sativa* using ILP and genomic SSR markers (Huang et al., 2010). The developed *DcS*-ILPs showed discriminatory power comparable to that of dominant markers, e.g., DArT (Grzebelus et al., 2014). The values of Wright's fixation index which were significantly higher than zero, as well as the lower mean value of observed heterozygosity, indicated an excess of homozygous allelic states expected in advanced cultivars. *DcS*-ILP-based analysis of genetic structure of the studied accessions showed clear differentiation of wild and cultivated carrot, supporting earlier observations based on DArT, SSR and SNP genotyping (Cavagnaro et al., 2011; Iorizzo et al., 2013; Grzebelus et al., 2014). Bayesian clustering, on both accession and pre-defined population levels, revealed the presence of four gene pools that generally could be attributed to the shape of the storage root, namely: (1) Chantenay, (2) Danvers, (3) Imperator, and (4) Paris Market, and corresponding to their breeding history, as proposed by Banga (1963) (Figures 3B,C). Having said that, a substantial level of admixture was apparent for few investigated cultivars, possibly resulting from inter-type crosses aiming to derive an intermediate root morphology, e.g., longer or shorter roots. On the other hand, clear separation between the Paris Market type cultivars and the remaining three types confirms the postulated origin of the former from the Early Short Horn gene pool, opposed to Danvers and Chantenay types originating from the Late Half Long Horn gene pool. It is the first molecular evidence for a possible root-type associated structure of genetic diversity in western cultivated carrot. Nonetheless, a more extensive study ought to be conducted in order to substantiate this hypothesis. The results of PCoA were mostly consistent with Bayesian clustering indicating the presence of the above-mentioned genetic structure.

## Conclusion

In this study, we showed that the abundance of class II transposable elements may serve as a tool for relatively rapid and low-cost development of gene-derived molecular markers for effective use in carrot genotyping studies. *DcSto* insertion-derived ILP markers detect substantial variation among carrot plants of different origin and can be exploited in germplasm characterization and analysis of genome relationships. In addition, *DcS*-ILP markers directly reflect the variation within the genes and could be potentially useful in gene tagging and genetic map construction. ILP markers share many advantages of SSR markers, i.e., codominant nature, locus specificity and high reproducibility, but provide more convenient and rapid detection. To our knowledge, the *DcS*-ILP markers developed in this study are a novel set of publicly available transposon-based markers in the carrot.

## Author contributions

AM, DG, and KS designed the study; KS, AM, and GM developed *DcS*-ILP markers; KS performed the validation of candidate *DcS*-ILP markers and the assessment of genetic diversity; KS, DG, AM, and GM drafted sections of the manuscript; KS and DG prepared the final version of the paper. All authors read, reviewed and approved the manuscript.

## Funding

The research was financed from funds for basic research on crop improvement granted by the Polish Ministry of Agriculture and Rural Development in the years 2014–2016.

### Conflict of interest statement

The authors declare that the research was conducted in the absence of any commercial or financial relationships that could be construed as a potential conflict of interest.
